# NF-κB, the Importance of Being Dynamic: Role and Insights in Cancer

**DOI:** 10.3390/biomedicines6020045

**Published:** 2018-04-17

**Authors:** Federica Colombo, Samuel Zambrano, Alessandra Agresti

**Affiliations:** 1 Division of Genetics and Cell Biology, San Raffaele Scientific Institute, 20132 Milan, Italy; colombo.federica@hsr.it; 2 Department of Electronics, Information and Bioengineering, Politecnico di Milano, 20133 Milan, Italy; 3 Vita-Salute San Raffaele University, 20132 Milan, Italy

**Keywords:** NF-κB, dynamics, live cell imaging, microfluidics, cancer, transcription

## Abstract

In this review, we aim at describing the results obtained in the past years on dynamics features defining NF-κB regulatory functions, as we believe that these developments might have a transformative effect on the way in which NF-κB involvement in cancer is studied. We will also describe technical aspects of the studies performed in this context, including the use of different cellular models, culture conditions, microscopy approaches and quantification of the imaging data, balancing their strengths and limitations and pointing out to common features and to some open questions. Our emphasis in the methodology will allow a critical overview of literature and will show how these cutting-edge approaches can contribute to shed light on the involvement of NF-κB deregulation in tumour onset and progression. We hypothesize that this “dynamic point of view” can be fruitfully applied to untangle the complex relationship between NF-κB and cancer and to find new targets to restrain cancer growth.

## 1. Introduction: NF-κB, the Importance of Being Dynamic

NF-κB is a family of transcription factor dimers formed by the combination of p50/NFKB1, p52/NFKB2, c-Rel, p65/RelA and RelB. These transcription factors are involved in development, inflammation and immune response and play a crucial role in chronic inflammation as well as cancer initiation and progression [1,2,3,4].

**Figure 1:**
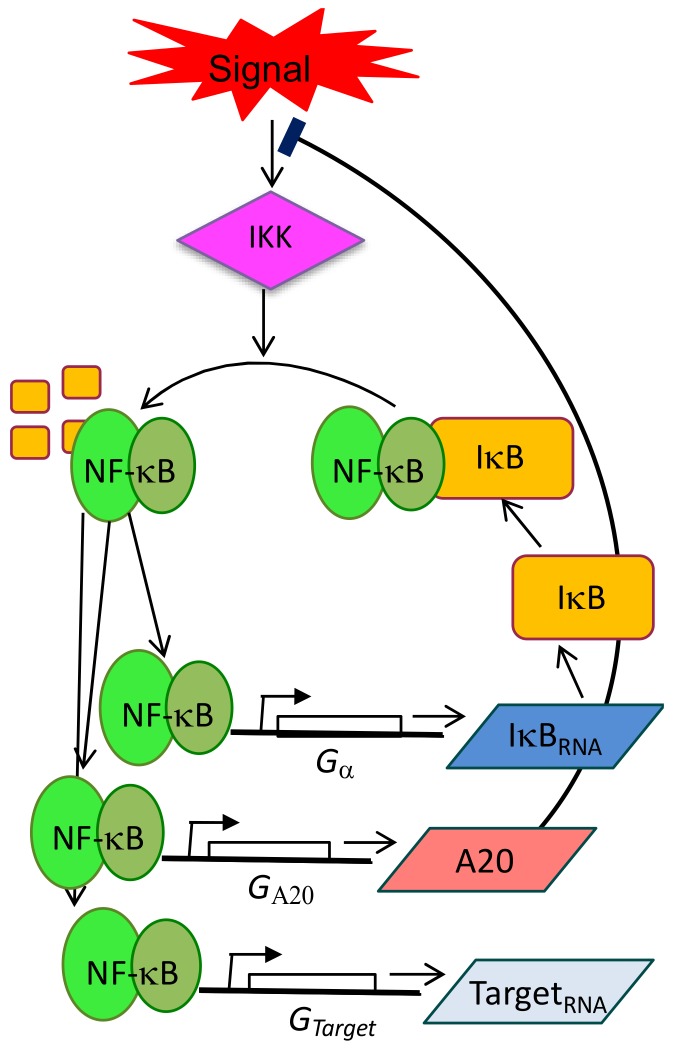
Schematic representation of NF-κB circuitry, with particular emphasis on the negative feedback loops controlled by IκBs and A20 proteins. Such circuitry is extended to the control of a set of target genes IκBα, A20 and “Target” genes, suggesting that NF-κB dynamics can directly operate the dynamics of the transcriptional output.

In physiological conditions, NF-κB homo- and heterodimers are constrained in the cytoplasm by the interactions with IκB inhibitors (existing in three isoforms IκBα, IκBβ, IκBε) [5]. Different extracellular signals, from inflammatory cytokines like the Tumour Necrosis Factor TNF-α, to the component of the bacterial wall (Lipopolysaccharide, LPS) activate mainly three NF-κB family members (RelA/p65, p50 and cRel) that translocate simultaneously into the nucleus as a result of repressors degradation modulated by IKK kinases activity. Once in the nucleus, NF-κB activates 200–500 target genes, including those coding for IκBs, which shape the delayed inhibitory feedback loop in the system. Newly translated IκBs enter the nucleus, bind to NF-κB and relocate the complex in the cytoplasm [6]. A second independent negative feedback is represented by the ubiquitin-editing enzyme TNFAIP3, historically known as A20 (Figure 1). Not to forget, the described canonical pathway is flanked by an alternative one that is activated through different receptors (BCR, BAFFR, CD40, RANK, LTβR [7]) and their ligands, recruits the other two NF-κB family members, p52 and cRel and activates transcription. However, the mentioned alternative players as well as the interactions between the canonical and the alternative pathways have rarely been considered in dynamics studies and this underscores our superficial knowledge on the NF-κB system in normal and cancer tissues.

The presence of at least two strong negative feedback loops provides both system flexibility and a tight dynamical control of the response to external stimuli. As a consequence of this sensitive wiring, a constant stimulation with inflammatory stimuli translates in oscillations of nuclear NF-κB concentrations that were first observed via microscopy almost 15 years ago [8] and then in a variety of studies (revised in [9]). Importantly, it has been demonstrated that such oscillations are fundamental to modulate gene expression [10,11,12,13] in a functionally relevant way [14], as we will discuss in detail below.

Research in the past recent years using single-cell dynamics approaches has shown that the NF-κB circuit is one example among several that have been found to have rich dynamics [15]. Such list includes also the tumour-suppressor pro-apoptotic p53 whose switch from oscillating to sustained dynamics is able to determine the cell fate [16]. These approaches have also shown that the dynamics of NF-κB in single cells are quite heterogeneous according to each cell’s susceptibility and to the inherent stochasticity of the system [8,14,17,18]. This heterogeneity was averaged out and hence went unnoticed in the first biochemical studies, where NF-κB appeared almost as non-oscillating [5,19]. Further insights on the role of NF-κB dynamics have also been provided by microfluidic cell culture technologies [20], able to reproduce, at least in part, complex time-varying signals that single cells receive from the environment. Importantly in this context, data-driven mathematical modelling of the NF-κB system [8,14,19,21] has reached a considerable sophistication and has contributed to integrate in a quantitative way all the information. This is a topic of great interest, but we will not review for sake of space here, although we will further outline its importance later.

We will instead focus on the technological developments and the fundamental experimental insights on the role of NF-κB, since we believe that they might have a transformative effect on how NF-κB involvement in cancer is studied. The availability of cancer genomic and proteomic datasets has allowed the identification of many genes in both signalling pathways that are silenced, overexpressed or expressed below the normal threshold [22]. Since NF-κB inhibits apoptosis and fosters proliferation, the deregulated expression of these genes become central in cancer pathobiology [3,23,24]. However, this again provides a quite static view of the role of NF-κB in cancer, that might be enriched by new insights on its role as a dynamic player.

We aim at revising and highlighting recent findings on the output of NF-κB dynamics, with the cutting-edge approaches that have made them possible. We believe that the increasing importance of NF-κB dynamics can impact on our understanding of its involvement in cancer and would potentially help identify new and maybe more specific targets for cancer therapies. Before starting our description, in the next section we will briefly review the different levels at which NF-κB regulation can be affected in cancer.

## 2. NF-κB and Cancer

Many possible combinations of intrinsic and extrinsic factors account for NF-κB deregulation in a wide variety of cancers. “Intrinsic factors” are represented by mutations or epigenetic alterations either in the coding and regulatory sequences of genes belonging to the NF-κB pathway, or in their targets. The NF-κB subunits RelA/p65, p52 and RelB are highly expressed and chronically activated in almost 80% of the tumours [25,26,27] and thought to fosters NF-kB anti-apoptotic and pro-proliferative activities as well as contribute to all the remaining hallmarks of cancer [28,29]. Gain-of-function and loss-of-function mutations affecting regulatory genes like A20, IκBα and CYLD (TNFAIP3, Nfkbia, Cyld Lysine 63 Deubiquitinase, respectively) result in the activation of both the classical and alternative NF-κB pathways as evaluated taking into account the processing rate of p50 and IκBα, the phosphorylation of IκBα and p65/RelA, the nuclear localization of NF-kB dimers and, last and extremely relevant, their DNA-binding activity together with the expression of NF-κB target genes [26,30]

Even though the abundance of mutations would argue for a prominent role of non-canonical NF-κB signalling in cancer pathogenesis, a tight crosstalk between the two pathway leads to a diffuse activation involving also the canonical one [24,30,31,32,33,34]. Indeed, the detection of mutations in specific genes cannot predict the global outcome for NF-κB activation, nor whether the crosstalk between the canonical or alternative pathways might result blocked or fuelled [35] thus obliterating the relevance of Exome, DNA or RNA sequencing at population or tissue level. 

The association of NF-κB activity and cancer has been exclusively provided at population level and inferred from static pictures of proteins localizations and dosage. Importantly, these data do not positively contribute to the prognosis since the overexpression of one of the NF-κB proteins can be either favourable or unfavourable in different tumours [36,37,38,39,40] as happens for p52/NF-κB2 in melanoma or renal and colorectal cancers, respectively (https://www.proteinatlas.org) or for the tumour suppressor TP53 in endometrial cancer (favourable) and prostate cancer (unfavourable) [41].

The term “extrinsic factors” collectively includes the autocrine/paracrine signalling provided by soluble molecules produced by the many cell types that add up to the signals originating from cell-to-cell interactions in the ever-changing inflammatory microenvironment [42] which of course modulates the degree and extent of NF-κB activation in all the cells. This complexity points to the need of a deeper understanding on how intrinsic and extrinsic factors interact to produce NF-κB deregulation in cancer [43].

As an example, regarding the complex tumour-environment interactions [44], two recent studies on NF-κB signalling in stromal fibroblasts in colitis-associated models of cancer showed that the inhibition IKKβ, a major activator of the canonical NF-κB pathway, results in either the promotion or the repression of cancer development. The comparison of the procedures to knock IKKβ out, identified the different time schedule of IKKβ genetic ablation [45] as the most probable culprit and highlighted the necessity of proper and time—defined dynamic interplay between intrinsic and extrinsic factors in tumour onset, progression and response to therapies.

A paradigmatic example for NF-κB dependent tumour-stroma interactions is represented by Multiple Myeloma (MM). In MM, constitutive NF-κB activity due to mutations in both pathways [23] critically contribute to tumour progression in the bone marrow (BM) microenvironment. Indeed, NF-κB in plasma cells (PCs) is activated by SDF1 (Stromal Derived Factor-1 or CXCL12) released by stromal cells in the BM. Activated PCs, in turn, produce IL-6, an inflammatory cytokine that induces the stromal components to produce more SDF-1. This positive feedback loop, entirely based on NF-κB activity and crucial for physiologic PCs survival and proliferation, leads to NF-κB hyperactivation in both cell types and drives cells toward the malignant phenotypes independently from the stromal support [46].

An additional example comes from the establishment of bone metastasis as a result of the reciprocal engagement between tumour cells and normal host cells of the bone microenvironment (for example, osteoclasts, stromal cells, vascular cells, etc.). In particular, cancer cells activate the production of the NF-κB regulated paracrine factor RANKL (Receptor Activator of NF-κB Ligand) in stromal cells which in turn pathologically increases osteoclasts activity and bone resorption, both hallmarks of bone metastases and critical for metastatic establishment and progression [47,48]

Overall, this non-exhaustive list of examples highlights how NF-κB deregulation in cancer is extremely diversified, being the result of interactions of many players at many levels: mutations can occur in NF-κB proteins or in their target genes, either in the regulatory or in the coding sequences. Additionally, the resulting inflammatory or perturbed microenvironment might have an unpredictable contribution to progression. 

As mentioned above, several dedicated groups [8,10,11,12,14,18] have shown that NF-κB signalling dynamics at single cell level are very rich and the cell populations have heterogeneous features that have been overlooked using the static approaches. 

In our opinion, the recently developed techniques could be instrumental to untangle the complex interaction of intrinsic and extrinsic factors in cancer onset and progression. For these reasons in the next section we will highlight some of these techniques, summarize the main insights that have followed and try to draw connections to potential implications in the study of cancer.

## 3. Single-Cell Dynamics: Experimental Methodologies

### 3.1. Cell Systems and Fluorescent Tags

The availability of fluorescent labels for proteins triggered the investigation on single cell dynamics. The easiness of use made cancer-derived cell lines the preferred model for these studies. For example, in the pioneering work of Nelson et al. [8], SK-N-AS cells (human S type neuroblastoma cell line) and HeLa cells (human cervical carcinoma cell line) were stably transfected with a vector expressing RelA fused at the N terminus of a red fluorescent protein [8,10]. An N-term tagged pEGFP-RelA vector have also been used in a number of studies using HeLa cells [12,49]; liver hepatocellular carcinoma HepG2 cells [50] and in breast tumour cell lines [51].

A first note of caution is mandatory at this point. NF-κB dynamics and transcription profiles obtained from a cancer cell line will be informative only on that specific cell line, or on the specific cancer the cell line it has been derived from. In fact, phenotypic and genotypic changes during cancer development in vivo, or procedure for cell line isolation, in vitro, might have generated adaptive pressures on the sensitive NF-κB equilibrium that has further drifted away from the original one [52]. Therefore, it would be wise to dedicate attention at obtaining information on NF-κB dynamics also from the normal tissue/cell line counterpart, beside the tumour one. However, significant differences in dynamics might not emerge from the comparison until perturbations (activating stimuli) exacerbating hidden features are applied to cells. 

The wide heterogeneity in NF-κB responses among cell systems encountered in NF-κB literature might just reflect this technical approximation. We are still far from having a clear picture of *bona fide* physiological NF-κB dynamics and probably many flavours exist in unrelated cells and tissues. We would just need to understand how NF-κB is regulated in normal tissues.

Other popular non-tumour cell lines like RAW264.7, a murine macrophage-like line, have been used in some studies [53,54,55]. NIH-3T3 cells in which the RelA gene has been knock-out and its expression replaced by a transduced fluorescent RelA–DsRed fusion protein under control of the endogenous RelA promoter [9,56,57] have been used as well.

**Figure 2:**
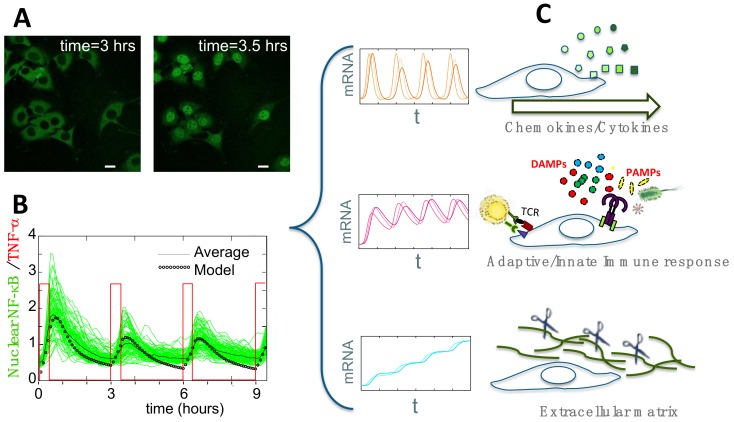
(**A**) Activation of GFP-p65 mouse embryonic fibroblasts upon stimulation. Untreated cells, left panel; cells stimulated with TNF-α for 30 min, right panel. Scale bar: 10µm. (**B**) Such activation can be modulated via a microfluidics device that delivers squared pulses of TNF-α (red profile). Synchronous oscillations from hundreds of cells can be measured (green lines) and compared with the averaged profile (black line) and with the dynamics predicted using a mathematical model (black dotted line). (**C**) Genome-wide gene expression profiling of the synchronized population that shows oscillations locked to the pulsed stimulus in B, revealed that genes can be clustered in three distinct dynamical patterns, each enriched in genes engaged in discrete cell functions.

The use of a fluorescently labelled p65 on a p65 knockout background would guarantee absence of interference between the endogenous and the exogenous versions of the protein—an issue that is seldom addressed properly. We must keep in mind, however, that p65/knock-out cells might have adapted their natural NF-κB signalling pathway and changed their phenotype during genetic manipulation procedures. Luckily enough, physiologic NF-κB expression level can be obtained by tagging the endogenous protein and for this specific reason a homozygous EGFP/RelA knock-in mouse was generated [58]. Fibroblasts (shown in Figure 2A) were derived from mouse embryos, immortalized and successfully employed in a number of works [11,14,18]. However, studies on primary cells from this mouse have not yet been published, presumably because they are more challenging to culture and the necessary procedures to isolate primary cells can activate them. As a reminder, the fluorescence intensity in homozygous knock-in cells is challenging low as expected from cells containing 25–30,000 tagged p65 proteins (unpublished data). We must be aware that the transient RelA expression from constitutive promoters produces a fluorescent signal that is at least 100-fold brighter than the fluorescence in p65 knock-in cells. Therefore, the sensitive NF-κB pathway might dramatically react to overexpression by reorganising itself.

The new CRISPR/Cas9-based gene-editing techniques [59] are expected to provide an important methodological leap forward in this context, allowing to tag NF-κB genes in their endogenous loci in cell types of choice, including cancer cells. This approach has been well validated to knock-out selected genes by introducing indel mutations in the coding frame. Actually, it still is in its infancy when a coding sequence must be inserted in frame with an endogenous gene. Unfortunately, although extremely useful to engineer tagged genes with physiological expression, this approach seems to be still in its infancy and the scientific community is looking forward to further and quick improvements [60]. In sum, different strategies have been utilized to fluorescently label NF-κB, each with its advantages and limitations that should be weighed when studying assorted aspects of NF-κB in tumour onset and progression.

Overall, the careful choice of the expression system for the tagged protein is necessarily a trade-off between sensitivity, specificity and physiological conditions that should also be weighed when studying the involvement of NF-κB in a given cancer type.

### 3.2. Experimental Observation and Quantification of Single-Cell NF-κB Dynamics

Once the cell line expressing NF-κB fused with a fluorescent protein is obtained, the next goal would be to extract high-quality data on NF-κB localization, that are able to represent well the expected heterogeneity as well as any features that would be blurred in population-level biochemical assays. 

Concerning quantitative microscopy, no gold reference exists to quantify NF-κB fluorescence at single cell level. With a fluorescence widefield microscope or with a confocal microscope with a well open pinhole [18], it is possible to get a 2D fluorescent signal coming from the whole cell thickness. This implies though that the layers of cytosol above and below the nucleus will contribute to the “nuclear intensity” of the signal. This is not a problem for adherent cells as fibroblasts, where such layers are very thin compared to nuclear thickness. However, this approach can be misleading when round cell, such as monocytes, are observed. In this case, acquire a confocal z-stack and reconstruct the geometry of the cells to estimate the total amount of NF-κB in each compartment would be the solution. A note of caution in this case suggests that z-scanning requires repetitive and invasive imaging, so photobleaching and photodamage might become an issue for cells health and signalling. 

A point of particular interest that is seldom discussed in detail regards the parameters that best describe NF-κB concentration in single cells. Being a transcriptional activator, the nuclear amount of NF-κB is probably the most informative one and different related magnitudes have been shown to be predictive of the transcriptional output [12,17,61]. However, in long time lapses, photobleaching plus variations of the focus and of laser intensity might alter the proportionality between the light emitted from a cell compartment and the protein content. That is why internally normalized measures are often used in the field and include ratios of nuclear-to-cytoplasm intensities [8,14,50,57], nuclear-to-total intensities [18] or average intensities [10,54]. As we reasoned elsewhere [18], the nuclear-to-total ratio is proportional to the total nuclear amount as long as the protein amount per cell is constant throughout the time lapse. This is taken as a given system parameter in mathematical models (see [62] and references therein) and its constancy has been shown at population levels for cells under TNF-α [14,19]. However, a recent work [54] showed the existence of a positive feedback loop that leads to an increase in the p65 total levels in macrophages stimulated with LPS, an increase that would be missed by using internally normalized measures. Hence intensity ratios, although able to eliminate distortions, should be used with awareness.

Finally, to extract information from these time-lapse experiments, most approaches require the segmentation of nuclei (based on either nuclear dyes such as Hoechst 33342 [14,18,55] or fluorescent histones [17,56]). Alternatively, tracks of nuclei position can be recorded without labelling before stimulation, when the nuclei are empty and hence easily visible but only in rather immobile cells [11,55]. Dedicated software propose the automatic segmentation of nuclei and cytosols to quantify single-cell NF-κB oscillations and can be found in a number of references [14,18,55]. However, in our experience a visual image inspection is always appropriate and common-sense to double-check software errors and technical flaws.

### 3.3. The Emerging Importance of Microfluidics Technologies

Microfluidic devices are becoming an important tool to gain further insight in the heterogeneity of NF-κB dynamics, potentially with single cell resolution under time-varying stimulation or multiple stimuli [63]. Both commercial or “custom-made” microfluidic devices are reported in the literature to study NF-κB dynamics [56,64,65,66,67,68,69]. A pioneering example of a microfluidic platform for mammalian cell culture is the so called “Cell Culture Chip,” with 96 individually controlled chambers that can support the growth of 1–1,000 cells [64]. The device is made of two layers: the “flow layer”, which contains cells and reagents and the automatically controlled “control layer” that creates microfluidic valves. This device was used to study NF-κB dynamics in 3T3 cells expressing p65-DsRed protein under different doses of TNF-α [17] and to analyse the dynamics under periodic (sawtooth-like) TNF-α pulses [56,57]. A commercial alternative is the CellASIC^®^ ONIX Microfluidic Platform (EMD- Millipore, 2018 Merck KGaA, Darmstadt, Germany) used to study NF-κB dynamics in GFP-p65 knock-in MEFs under different periodic stimulations [14] (see Figure 2B). Time varying external stimuli can also be obtained using a simple “custom-made” microfluidic chip, where pulses can be generated by manipulating the relative heights of two reservoirs connected to the cell chambers and containing the medium plus the stimulus/drug and the medium alone [49,66].

Microfluidics can also be employed for other scopes, as for the real-time imaging of host-pathogen interactions using devices able to trap host cells and put them in contact with bacteria or LPS [53]. Interestingly, the CellASIC^®^ ONIX system was used to generate transient interactions between pathogenic bacteria (*Salmonella typhimurium*) and mammalian cells. Unexpectedly, the infected cells showed impaired NF-κB response upon a second challenge with bacteria or inflammatory cytokines [67].

Microfluidic devices have also been used to perform other assays and complement the information obtained from single cell microscopy. In example, “Imstain” is a microfluidic system built for culturing, stimulating, fixing and immunostaining of cells [65]. Furthermore, the device described in [55] adapted a single-cell trapping for RNA-sequencing to make it also compatible with live cell imaging (before the sequencing).

Taken together, microfluidic technologies allow a more accurate and physiologic manipulation of cells and their environment for live cell imaging, as well as the combination with complementary biochemical assays. Microfluidic approaches then can become a fundamental tool to study in vitro the complex crosstalk between cancer and stroma, that we have just started to unravel.

## 4. Single-Cell Dynamics: Main Insights on NF-κB Regulation of Gene Expression

### 4.1. Single-Cell NF-κB Dynamics: Main Insights

We have seen so far that a variety of techniques have been used to quantify single-cell NF-κB dynamics. Such techniques have been applied in a myriad of papers appearing in the last 15 years and gave rise to important insights on NF-κB and its role. Here we will attempt to summarize some of the main features emerging from these analyses. 

***NF-κB oscillates***: probably the most acknowledged feature of NF-κB dynamics is the presence of oscillations upon external stimuli. This was predicted by mathematical models of the NF-κB regulatory pathway [19] and later verified using microscopy approaches in single cells [8]. The oscillatory dynamics has later been observed for different cell types [11,14,18,50,56]. The period of the oscillations is widely accepted to be close to 90 min. The oscillatory pulses can be more or less spiky, as predicted by mathematical models but peak shapes might be due either to the nature of quantifiers used to asses NF-κB dynamics or to the cell type used [18]. 

***Heterogeneity in the response***: the fraction of cells responding depends on the dose, as well as the amplitude of the oscillatory peaks and in general the dynamics can be heterogeneous in the population of cells [8,17,18]. However, in some of the cells responsive to TNF-α, such as the widely used HeLa cells, sustained oscillations are infrequent upon inflammatory stimulation [8,12]. The type of dynamics observed can be determined by the stimulus applied; for example, NF-κB nuclear localization exhibits oscillatory dynamics when 3T3 cells are stimulated with TNF-α but a stable nuclear accumulation occurs when cells are stimulated with LPS [68]. Moreover, Lee et al. demonstrated that molecules of TNF-α produced during LPS stimulation lead to a secondary paracrine NF-κB activation in the cell neighbourhood [69]. Oscillations are not present either in LPS-stimulated murine primary pEGFP-p65 knock-in macrophages (unpublished data) and in the murine monocytic cell line RAW264.7 stimulated with LPS [54]. Unexplained discrepancies on different dynamics in similar cell systems suggest that our understanding of NF-κB regulation requires further improvements. In particular the debate is still open on whether the NF-κB oscillations are sustained, meaning that cycles would go on as long as the cells are under stimulus, as shown for 3T3 p65 knock-out fibroblasts expressing exogenous GFP-tagged p65 [56], or damped, meaning that the cycling oscillatory peaks tend to decrease their amplitude until complete disappearance, as shown for immortalized MEFs in [14]. We feel confident to exclude that dampening might be due to TNF-α degradation/internalization, since in microfluidics devices cells are grown in small chambers where the stimulus is continuously renewed. 

***Mathematical models***, indeed, predict the presence of both sustained and damped oscillations upon the same stimulation that depend on how the negative feedbacks of the NF-κB regulatory system are tuned [14]. This was already suggested through biochemical approaches when the regulatory circuitry of NF-κB was unveiled [19]. Hoffmann and collaborators showed that the knockouts for the inhibitors, IκBβ and IκBε led to more pronounced, or more synchronized oscillations, than in wild type cells. Indeed, the relative intensity and timing of the feedback can impact on the overall dynamics and might play a role in maximizing population heterogeneity in the oscillations [21]. When oscillations were first described in single cells [8], mathematical modelling also suggested that higher expression level of NF-κB could be responsible for the dampening of the oscillations [70] but this feature was not confirmed experimentally [71]. Overall, we can anticipate that even subtle variations in players of the NF-κB regulatory circuitry can lead to diverse dynamics and such diversity represents the necessary adaptive flexibility to coordinate diverse biological functions, a hypothesis that has recently started to be explored systematically using synthetic NF-κB genetic circuits [72].

***Microfluidics***: On the other hand, microfluidics allows to explore more complex questions, such as how the NF-κB system responds to time-varying stimuli (mimicking the dynamic environment where the inflammatory response takes place). Pulsatile stimuli were indeed shown to produce a synchronous oscillatory response in the cell population [10]. Using cells showing sustained oscillations upon chronic stimulations, Kellog et al. recreated a sawtooth-like periodic profile of TNF-α stimulation with a home-made microfluidic chip and found that oscillations synchronised to some – not every – stimulation frequencies, a behaviour that could be referred to as “entrainment” [56]. Mathematical models suggest that noise can widen the range of acceptable synchronization modes (i.e. the acceptable ratio between oscillation frequency and the frequency of the external perturbation) and in turn leads to the hopping between them [57]. Damped NF-κB oscillations instead seem to adapt quickly to a wide range of frequencies without any preferred one and with no memory of the synchrony [14]. HeLa cells have also been found to respond quite synchronously as extremely damped oscillators to pulses of external stimuli [73].

Overall, the use of these techniques enlightens how the NF-κB system is able to adapt its activation dynamics to the time-varying external stimuli, although with subtle differences between cell types and stimuli. This is of critical importance in the study of cancer cells where the NF-κB circuitry is deranged. Furthermore, the use of microfluidic devices is now allowing to explore how NF-κB responds to complex environmental cues and this can also lead to important insights on the role of NF-κB in the interaction between the cancer cells and their microenvironment.

### 4.2. Connecting Dynamics to Transcription

The molecular control of transcription by NF-κB has been the subject of intensive research [61] but the combination with data from single-cell dynamics data has brought novel interesting insights. It was quickly identified that in oscillatory populations target genes were expressed with different dynamics, which included early, intermediate and late-responding genes [8,12]. By constraining RelA in the nucleus by means of LMB or CHX (Leptomycin or Cycloheximide, respectively) the transcriptional output of early and late gene expression was severely impaired or enhanced, respectively, indicating that the oscillatory dynamics is a fundamental ingredient to produce the timely transcription of target genes [11]. Other works showed that the amount of mRNA of NF-κB targets in the population would nicely scale with the area under the first peak of nuclear activation of the fraction of cells responding to the stimulus [17]. 

Time varying external signals also provide insights in this context. The use of a pulsatile stimulation showed that the dynamics of distinct genes could vary depending on the amplitude and the frequency of the pulsatile input. Thanks to microfluidics, this kind of studies have been extended. Microfluidics-generated sawtooth-like stimulation of cells, able to entrain sustained oscillatory NF-κB dynamics, would also lead to an increased transcriptional output [56]. A similar experimental setup based on squared pulses of TNF-α allowed us to show [14] that synchronous NF-κB oscillations translate into three transcriptional outputs: a fraction of genes would oscillate in sync with the nuclear-to-cytoplasm NF-κB translocations; a second group steadily accumulated, while intermediate dynamics were also possible (Figure 2C). Each pattern of gene expression was enriched in genes with similar biological functions, underlining the connection existing between dynamics and function of genes under the control of NF-κB [14] (Figure 2C). Interestingly, a recent microfluidics study employing single-cell culture chambers connected NF-κB dynamics to single cell transcription. Three main patterns of NF-κB dynamics under LPS activation were identified and each pattern correlated with a specific pattern of relative abundance of the common transcripts [55]. Finally, the use of a precise microfluidics device has shown that differences in the stimulus duration can lead to the activation of either pro or anti- apoptotic transcriptional programs, which impact directly on cell fate [49]. 

Considering the heterogeneity in gene expression even in a population of identical cells [15,74], it is important to characterize how single-cell NF-κB dynamics modulates (amplifies or tames) this stochasticity. The first works on single-cell NF-κB dynamics [8] indeed linked NF-κB dynamics to the translation of a fluorescently labelled IκBα reporter and confirmed what the model predicted: IκBα levels would oscillate synchronously with NF-κB nuclear localization but out of phase. A similar approach at single cell level showed the expression of a mCherry-reporter under the control of the TNF-α promoter (paradigmatic example of NF-κB controlled gene) whose accumulation appeared coordinates with the activation of NF-κB dynamics [54]. By using RNA-FISH a recent study has correlated the expression of different genes in single cells with dynamical features of NF-κB activation, finding that nuclear concentration fold change (and not the area under the curve of the activation peak) is the best predictor for the number of RNA copies produced [12]. Only through a more thorough characterization—including a wider variety of cell types—we will be able to understand how NF-κB modulates gene expression in single cells. 

## 5. Conclusions

Cancer is a complex system in which intrinsic and extrinsic factors contribute to its growth, progression and disguise from the host immune defence. However, these factors are never at the equilibrium, rather they are constantly evolving both in number and intensity, over time. 

In the past years NF-κB dynamics emerged as key regulators of cell life and death. This family of transcription factors, which in healthy tissues controls tissue homeostasis, responds to external stimuli and coordinates cell growth and differentiation, is often deregulated in cancer cells. A deranged signalling represents an important source of variability and heterogeneity in cancer growth that blunts therapeutic efforts.

In this review, we thus described the cutting-edge technological approaches that contributed to these novel insights. 

**Figure 3:**
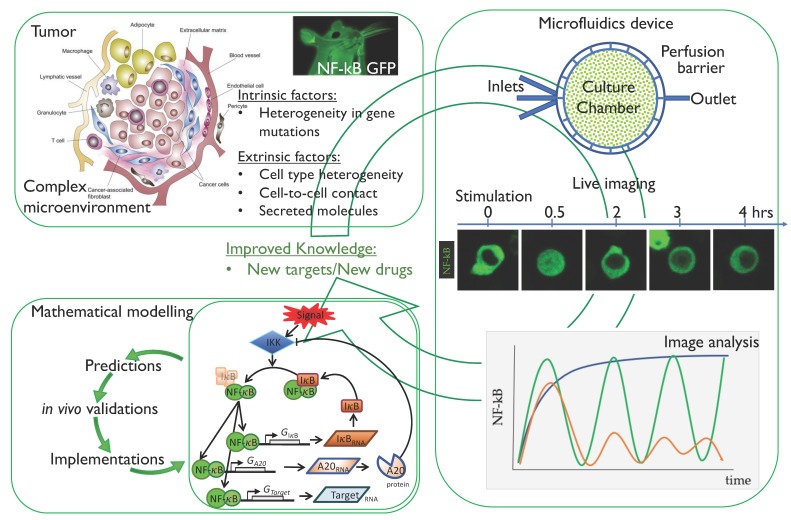
Workflow for the analysis of NF-κB dynamics to improve our knowledge in tumour biology. Starting from the tumour microenvironment [75], the intrinsic factors (mainly gene mutations in cancer cells) and extrinsic factors (cell to cell contact and secreted molecules) can be untangled and analysed by means of live imaging of cells expressing fluorescent NF-κB. In particular, microfluidics can help in recreating the external signals found in vivo. Finally, mathematical modelling represents a fundamental tool to fit live imaging data and to make predictions on NF-κB dynamics when either extrinsic, intrinsic or both components are modulated. The whole set-up may eventually improve our knowledge on tumour biology with the final aim of finding new molecular targets for pharmacological intervention.

In our opinion, the knowledge about the relationships between NF-κB regulation and cancer could be greatly enhanced by live cell imaging and microfluidics technologies described so far by following the workflow summarized in Figure 3. In fact, a dissection of intrinsic from extrinsic components causing NF-κB deregulation in cancer, should provide a clearer picture and would potentially help identify new and maybe more specific targets for cancer therapies. 

This is actually a difficult task that can be accomplished only by the joint effort of scientists in the field taking advantage of tools from the System Biology field. Mathematical and computational models are increasingly used to help interpret dynamic data from cell biology and imaging experiments. Importantly, mathematical simulations of complex biological processes are best fit to generate hypotheses and suggest experiments to further challenge and validate the model. 

Circular iterations of model implementation will allow more accurate predictions, (Figure 3) and can pave the way to fundamental new insights in NF-κB regulation in cancer cells.
